# DPP7 as a Potential Therapeutic Marker for Colorectal Cancer

**DOI:** 10.7150/jca.93112

**Published:** 2024-08-19

**Authors:** Li Ma, Hailang Yang, Shuwei Wu, Chunliang Wang, Jinhong Mei

**Affiliations:** 1 Department of Pathology, The First Affiliated Hospital of Nanchang University, Nanchang, China.; 2 Institute of Molecular Pathology, Nanchang University, Nanchang, China.; 3 Department of Urology, The First Affiliated Hospital of Nanchang University, Nanchang, China.; 4 Department of Neurosurgery, The First Affiliated Hospital of Nanchang University, Nanchang, China.

**Keywords:** DPP7, Colorectal cancer, Prognosis, Immune evasion

## Abstract

**Background:** Dipeptidyl peptidase 7 (DPP7) is overexpressed in various tumors, but its role in colorectal cancer (CRC) remains unclear. Study the Impact of DPP7 on malignant progression and tumor immunity in CRC.

**Methods:** We utilized Tumor Immune Estimation Resource 2.0 (TIMER2.0) and The Cancer Genome Atlas (TCGA) analyses to assess the expression of DPP7 in tumors and validated it through immunohistochemistry and immunoblotting. Additionally, we investigated the relationship between DPP7 and immune cell infiltration using single-sample Gene Set Enrichment Analysis (ssGSEA) analysis. Finally, the impact of DPP7 on cell proliferation, invasion, migration, and immune cell function in the tumor microenvironment was confirmed through cell experiments and animal studies.

**Results:** DPP7 is highly expressed in CRC, and high expression of DPP7 is associated with poor prognosis. Cell experiments demonstrate that overexpression of DPP7 enhances the proliferation, migration, and invasion capabilities of colorectal cancer cells both *in vitro* and *in vivo*. Immune infiltration analysis and co-culture results indicate that overexpression of DPP7 suppresses the immune cell's cytotoxic function against tumors in the tumor microenvironment.

**Conclusions:** DPP7 promotes the malignant potential of colorectal cancer cells and inhibits tumor immune function, thereby promoting the progression of colorectal cancer.

## Introduction

CRC, one of the most prevalent malignancies globally, has been a subject of continuous concern^1^. According to the 2020 World Health Organization (WHO) statistics, CRC ranks as the third leading cause of cancer-related deaths in both men and women worldwide. Specifically, the mortality count for male CRC patients globally stands at 515,637 individuals, while for females, it is 419,536, placing it at the third position for incident cancers and the second position for cancer-related mortality^2^. Disease recurrence and metastasis after surgery are significant contributors to the unfavorable prognosis of CRC^3^. Hence, investigating the underlying mechanisms of CRC and identifying novel therapeutic targets is paramount for improving patient outcomes. This study aims to delve into this issue and provide fresh insights for future treatment modalities.

Dipeptidyl peptidases (DPPs) are a class of aminopeptidases primarily responsible for influencing the stability of proteins by cleaving peptide bonds at the ends of peptide chains. The DPP family encompasses numerous members, including DPP1, DPP2, DPP3, DPP4, DPP5, DPP6, DPP7, DPP8, DPP9, DPP10, and DPP11, among others. These members exhibit functional distinctions and play crucial roles in regulating cellular metabolism and signaling cascades, among other vital biological processes. Research on the DPP family has shown that high expression of DPP1 and DPP3 is associated with lymph node metastasis and invasion of CRC^4^ ^5^. Further studies have revealed that DPP1 overexpression promotes the recruitment of myeloid-derived suppressor cells and tumor-associated macrophages by upregulating CSF1 expression, thereby facilitating CRC metastasis^4^. Additionally, Hederacolchiside A1, derived from sea cucumbers, can downregulate DPP1 expression, inhibiting the growth of CRC cells and inducing cell cycle arrest^6^. Downregulation of DPP3 expression can suppress the proliferation of CRC cells and upregulate apoptosis-related proteins Caspase3 and Caspase8, promoting tumor cell apoptosis^5^, ^7^. DPP4, a glycoprotein located on the cell surface, is uniformly expressed on the surface of primary and metastatic CRC cells. Membrane expression of DPP4-positive cancer cells is associated with increased tumor invasion and chemotherapy resistance^8^. Additionally, DPP4 is present in the serum of cancer patients, with higher levels in circulating blood observed in metastatic CRC patients, suggesting its potential as an early diagnostic and prognostic marker for CRC 9. MMP1 induces the expression of vascular endothelial growth factor receptor 2 (VEGFR2) and promotes endothelial cell proliferation. MMP1 promotes tumor-associated angiogenesis and metastasis by regulating DPP4 expression 10. DPP9 is aberrantly expressed in leukemia, non-small cell lung cancer, and serous ovarian cancer tumors 11-13,44-46. Downregulation of DPP9 expression weakens CRC cells' viability granules and increases tumor cells' sensitivity to chemotherapy^14^. Studies have indicated that DPP7 participates in the survival of resting lymphocytes, with DPP7 inhibition in these lymphocytes leading to apoptosis^15^.

DPP7 plays a crucial role in various cancers. Studies have shown that individuals with higher DPP7 activity typically exhibit a poorer prognosis in chronic lymphocytic leukemia (CLL) patients. In comparison, those with lower DPP7 activity tend to have a more favorable prognosis^16^. This research provides a basis for using DPP7 apoptosis assays as a feasible method for assessing the prognosis of CLL patients. It reveals the potential role of DPP7 in the development of CLL^17^.

Furthermore, studies have indicated that in hepatitis B virus-infected liver cancer cells, DPP7 expression levels increase, and the integration of the hepatitis B virus with DPP7 is closely related to the process of cell apoptosis^18^. However, bioinformatic research has found that high DPP7 expression levels are associated with a better prognosis in breast cancer patients, suggesting that DPP7 may function in a tissue-specific manner in different cancers^19^.

While high DPP7 expression levels have been identified as a significant prognostic indicator for poor outcomes in CRC patients^20^, the biological role of DPP7 in CRC and its relationship with the immune system remains enigmatic.

This study leveraged immunohistochemical data to assess DPP7 expression levels in CRC and established its relevance to patient prognosis. Our investigation validated our findings using the TCGA dataset and incorporated immunohistochemical data to comprehensively explore the potential role of DPP7 in CRC. Initially, we compared DPP7 expression levels in tumor tissues and adjacent non-cancerous tissues within the TCGA dataset. We investigated the relationship between DPP7 expression and clinical pathological features, as well as its prognostic value. Additionally, we conducted analyses to assess the association between DPP7 expression and immune cell infiltration and checkpoint markers, shedding light on the interplay between DPP7 and the immune system. Moreover, co-culture experiments of tumor cells with immune cells confirmed the impact of DPP7 expression in tumor cells on immune cell function.

Furthermore, Gene Set Enrichment Analysis (GSEA) was employed to investigate which signaling pathways might be modulated by DPP7 overexpression. Finally, a series of experiments were conducted to investigate the effects of altering DPP7 expression on tumor cell proliferation, invasion, and migration. These results were further substantiated through animal experiments. In summary, our findings lead us to conclude that DPP7 may be a potential prognostic biomarker for colorectal cancer. The aberrant upregulation of DPP7 expression could enhance tumor malignancy and promote immune evasion in colorectal cancer.

## Methods

### Patients and data collection

Broad-spectrum expression data for DPP7 were obtained from TIMER2.0 (http://timer.cistrome.org/)^21^, RNA-seq data processed by the STAR workflow from TCGA databases (https://portal.gdc.cancer.gov) were downloaded and curated for TCGA-COAD and TCGA-READ projects. FPKM-formatted data and clinical information were extracted. Perform differential expression analysis of DPP7 among CRC patients with different clinical characteristics using the R statistical environment (version 4.2.1).

Furthermore, we collected CRC tissue samples from 8 patients during surgical resection at the First Affiliated Hospital of Nanchang University. Corresponding normal colorectal tissue samples were also collected. These samples were discarded tissues removed after surgery, and we stored them in liquid nitrogen. These samples will be employed in subsequent experiments, including Quantitative Real-time PCR (qPCR) and Western blot analyses.

### Immunohistochemistry (IHC) analysis

We selected colorectal cancer paraffin blocks from 80 CRC patients at the First Affiliated Hospital of Nanchang University and corresponding paraffin blocks from 30 normal colorectal epithelial tissues for IHC analysis to assess DPP7 expression. These samples were obtained from the Specimen Center of the Pathology Department of the First Affiliated Hospital of Nanchang University, and all patients signed informed consent forms when surgically removing diseased tissues. Clinical information for these patients was also collected. All patient samples underwent histopathological diagnosis by two pathologists to ensure diagnostic accuracy. For the IHC analysis, we used the DPP7 antibody (AP61032, 1:300; abcepta, Jiangsu, China) as the primary antibody. Following previously published methods 22, Scoring was based on staining intensity (no staining, 0; weak staining, 1; moderate staining, 2; strong staining, 3) and the percentage of positive cells (1-25%, 1; 26-50%, 2; 51-75%, 3; >75%, 4). The staining scores ranged from 0 to 12, providing an assessment of DPP7 expression levels.

### Survival analysis

Based on the DPP7 expression data collected from TCGA and our immunohistochemical analysis results, we need to divide patients into the DPP7 high expression group and the DPP7 low expression group according to the expression of DPP7. The further grouping is as follows: There are a total of 698 CRC patient cases from TCGA. After removing patients without clinical prognosis data, 643 cases remain, which are divided into the DDP7 high expression group and the DPP7 low expression group according to the median expression of DPP7. There are 321 cases in the high expression group and 322 cases in the low expression group. The data from immunohistochemistry, the same as the TCGA grouping method, we divide them into the high expression group and low expression group according to the median immunohistochemical score. There are 39 cases in the high expression group and 41 cases in the low expression group. The subgroup prognosis grouping method of TCGA data is the same as before. We screened out 521 cases of COAD patients and 177 cases of READ patients. After excluding patients without clinical prognosis data, 477 and 166 cases were left, respectively. According to the median, there were 238 cases in the DPP7 high expression group, 239 cases in the DPP7 low expression group in COAD, and 83 cases in both the DPP7 high expression group and the low expression group in READ. In further analysis of the relationship between DPP7 expression and prognosis in patients with positive lymph node metastasis, we excluded patients with negative lymph nodes (N0) in the pathology report and included patients with positive lymph node metastasis (N1 & N2) in the prognostic analysis. There were 275 cases of N1 & N2 patients in CRC patients, and a total of 272 cases were included in the analysis after excluding cases without prognostic data. Further, according to COAD and READ, there were 194 cases of N1 & N2 patients in COAD, with 97 cases in both the DPP7 high expression group and the low expression group. In comparison, there were 78 cases of N1 & N2 patients in READ, with 39 cases in both the DPP7 high expression group and the low expression group. Finally, we performed an overall survival (OS) analysis using the 'survival' and 'survminer' functions within the R software package to assess the relationship between DPP7 expression levels and the survival status of CRC patients.

### Analysis of the correlation between immune infiltration and immune checkpoint gene expression

We employed ssGSEA^23^ as a research method to investigate tumor-immune interactions. This is a widely used research tool in various cancer types 24. Our study analyzed the correlation between DPP7 expression and eight immune cell infiltrations, which include B cells, CD8+ T cells, T helper cells, cytotoxic cells, natural killer (NK) cells, dendritic cells (DCs), regulatory T cells (Tregs), and macrophages.

Furthermore, we conducted correlation analyses for three immune checkpoint genes using R software, including programmed cell death 1 (PD-1, also known as PDCD1), programmed cell death ligand 1 (PD-L1, also known as CD274), and cytotoxic T-lymphocyte-associated protein 4 (CTLA4). These analyses were based on Spearman's rank correlation coefficient, helping us understand the associations between DPP7 expression and immune checkpoint gene expression.

### Gene set enrichment analysis

We employed GSEA^25^ as our method of choice. This approach involved comparing the high DPP7 expression group with the low DPP7 expression group using various gene sets from MSigDB. Through this study, we aimed to identify potential signaling pathways that DPP7 might regulate. To accomplish this, we utilized the 'clusterProfiler' package, which helped us further interpret and understand the biological significance of the enrichment analysis results.

### Cell culture and transfection

We obtained the human normal colon epithelial cell line NCM460, as well as human CRC cell lines HCT116, SW480, and SW620 from the Cell Resource Center of the Chinese Academy of Sciences in Shanghai. HCT116, SW480, SW620, and human normal colon epithelial cells NCM460 were cultured in high-glucose DMEM medium (Solarbio, Beijing, China) with the addition of 10% fetal bovine serum (Excell bio, Jiangsu, China), and maintained at 37°C in a 5% CO2 atmosphere.

HCT116 and SW480 cells were seeded in six-well plates at a density of 2x10^5 cells per well. Subsequently, we transfected small interfering RNA (siRNA) targeting DPP7 and control siRNA (si-NC) or overexpression plasmid into the cells using RiboFECTTM CP transfection reagent (RiboBio, Guangzhou, China) according to the manufacturer's instructions. The sequences of the siRNAs targeting DPP7 were as follows: siRNA-1: GAAGCGTTCCGACAGATCA, siRNA-2: CGTCTGGACCACTTCAACT. Additionally, we constructed shRNA for gene knockdown using the pLV3-U6-MCS-shRNA-EF1a-CopGFP-Puro vector. The shRNA sequence targeting DPP7 was CGTCTGGACCACTTCAACT.

### RNA extraction and qPCR analysis

We extracted RNA from the cells using an RNA extraction kit (Axygen, Silicon Valley, United States) and subsequently performed reverse transcription using the cDNA Synthesis Kit (Vazyme, Nanjing, China). Furthermore, we conducted qPCR using the fluorescent quantitative PCR instrument (TianLong, Xian, China) and 2×Fast SYBR Green qPCR Master Mix. The qPCR was performed in triplicate, and the results were analyzed using the 2^-ΔΔCT method. The qPCR cycling conditions were as follows: initial denaturation at 95°C for 1 minute, followed by amplification at 95°C for 15 seconds and extension at 60°C for 30 seconds, repeated for 40 cycles.

### Primer sequences

qPCR primer sequences:

DPP7, Forward: GATTCGGAGGAACCTGAGTGC,

Reverse: TCAACCACGGAAGCAGGATCT;

β-actin, Forward: TCTCCCAAGTCCACACAGG,

Reverse: GGCACGAAGGCTCATCA.

The primer sequences of DPP7 overexpression plasmid:

Forward: GCGGAAGGCGACATGGGCTCC.

Reverse: GTCCTGTGCTCAGAGGCTGAG.

### Western blot

To prepare the lysates of transfected cells and tissues, we used sodium dodecyl sulfate-polyacrylamide gel electrophoresis (SDS-PAGE) for protein separation, followed by transfer of the separated proteins to a polyvinylidene fluoride (PVDF) membrane. Subsequently, the membrane was incubated for 1 hour in a buffer containing 5% skim milk and maintained at 4°C overnight with the following primary antibodies: GAPDH (60004-1-Ig, 1:10000; Proteintech, Wuhan, China), DPP7 (AP61032, 1:500; abcepta, Jiangsu, China), E-cadherin (20874-1-AP, 1:10000; Proteintech, Wuhan, China), N-cadherin (22018-1-AP, 1:10000; Proteintech, Wuhan, China), Vimentin (10366-1-AP, 1:10000; Proteintech, Wuhan, China). This incubation was conducted on a shaker at 4°C.

Subsequently, the membrane was washed three times with Tris-buffered saline containing 0.1% Tween 20 (TBST) at room temperature. Next, the membrane was exposed at room temperature and incubated on a shaker for 1 hour with the appropriate secondary antibodies (anti-mouse for GAPDH, anti-rabbit for DPP7, E-cadherin, N-cadherin, Vimentin). The incubation temperature was maintained at 25°C.

Finally, we used chemiluminescent detection reagents (WBKLS0100, Millipore, Burlington, MA, USA) to scan and visualize the protein bands.

### Cell proliferation assay

To investigate the effect of DPP7 on CRC cell proliferation, we used the CCK-8 assay kit (Bioss, Beijing, China). Following si-RNA treatment, we seeded HCT116 and SW480 cells into 96-well plates, with 3000 cells per well. The cells were then cultured for 24, 48, 72, and 96 hours and incubated at 37°C for 2 hours with the CCK-8 solution, and the absorbance was measured at 450 nm.

Additionally, we used the EDU assay kit (Servicebio, Wuhan, China) to assess the impact of DPP7 on CRC cell proliferation. Again, following si-RNA treatment, we seeded HCT116 and SW480 cells into 96-well plates, with 10,000 cells per well. After culturing the cells to the normal growth phase, we performed staining according to the EDU assay kit's instructions. Subsequently, we observed and photographed the cells under an inverted fluorescence microscope (Zeiss, Oberkochen, Germany).

### Cell migration assay

To assess cell migration ability, we placed Transwell chambers in 24-well plates and seeded HCT116 and SW480 cells treated with si-RNA (5 × 10^4 cells per well) in the upper chambers. The upper chamber cells were cultured in serum-free DMEM, while the lower chamber contained DMEM with 20% fetal bovine serum. The entire system was then placed in a constant-temperature incubator at 37°C and cultured for 24 to 48 hours. Afterward, we fixed the cells with methyl alcohol, stained them with 1% crystal violet, wiped away the non-migrated cells, allowed them to air dry, and counted them using photography.

### Cell invasion assay

To assess cell invasion ability, we placed Transwell chambers in 24-well plates and pre-coated the bottom of the upper chamber with a solidified matrix. Subsequently, we seeded HCT116 and SW480 cells treated with si-RNA in the upper chamber, with 6 × 10^4 cells per well, and cultured them using serum-free DMEM. DMEM containing 20% fetal bovine serum was added in the lower chamber. The entire culture system was then placed in a constant-temperature incubator at 37°C and cultured for 72 to 96 hours. Afterward, we fixed the cells with methyl alcohol, stained them with 1% crystal violet, gently wiped away non-invading cells, and allowed the chambers to air dry. Finally, we observed the stained cells under a microscope and took photographs.

### Co-culture experiment with immune cells

Jurkat cell activation conditions: Cultured in a complete medium containing 10ug/mL PHA and 50ng/mL PMA for 24 hours.THP-1 cell activation conditions: Cultured in a complete medium containing 50ng/mL PMA for 24 hours. For co-culture, tumor cells and fully activated immune cells were seeded at a ratio of 1:4 into the upper and lower chambers of migration chambers with a pore size of 0.4um. After co-culturing for 48 hours, the viability of tumor cells or immune cells in the lower chamber was detected using the CCK8 assay. Additionally, the culture medium containing Jurkat cells was extracted to detect IL-2 concentration, and THP-1 cell protein was extracted to detect PD-1 expression. According to the co-culture conditions, single-cell smears of THP-1 cells were prepared, and PD-1 expression in THP-1 cells was observed using an upright fluorescence microscope (Zeiss, Oberkochen, Germany).

### Statistical analysis

When comparing two or more groups, we employed t-tests, one-way ANOVA, or two-way ANOVA for statistical analysis. The Wilcoxon Rank-Sum test and the Kruskal-Wallis test were utilized to analyze the relationship between DPP7 expression and clinical characteristics. We conducted univariate and multivariate Cox regression analyses to regress prognostic risk factors. A p-value less than 0.05 was considered statistically significant in all statistical analyses.

### Ethical approval

This study was approved by the Ethics Committee of the First Affiliated Hospital of Nanchang University, Ethics license number: (2022)CDYFYYLK(10-006); CDYFY-IACUC-202310QR011.

## Results

### DPP7 is upregulated in CRC

**Figure 1:**
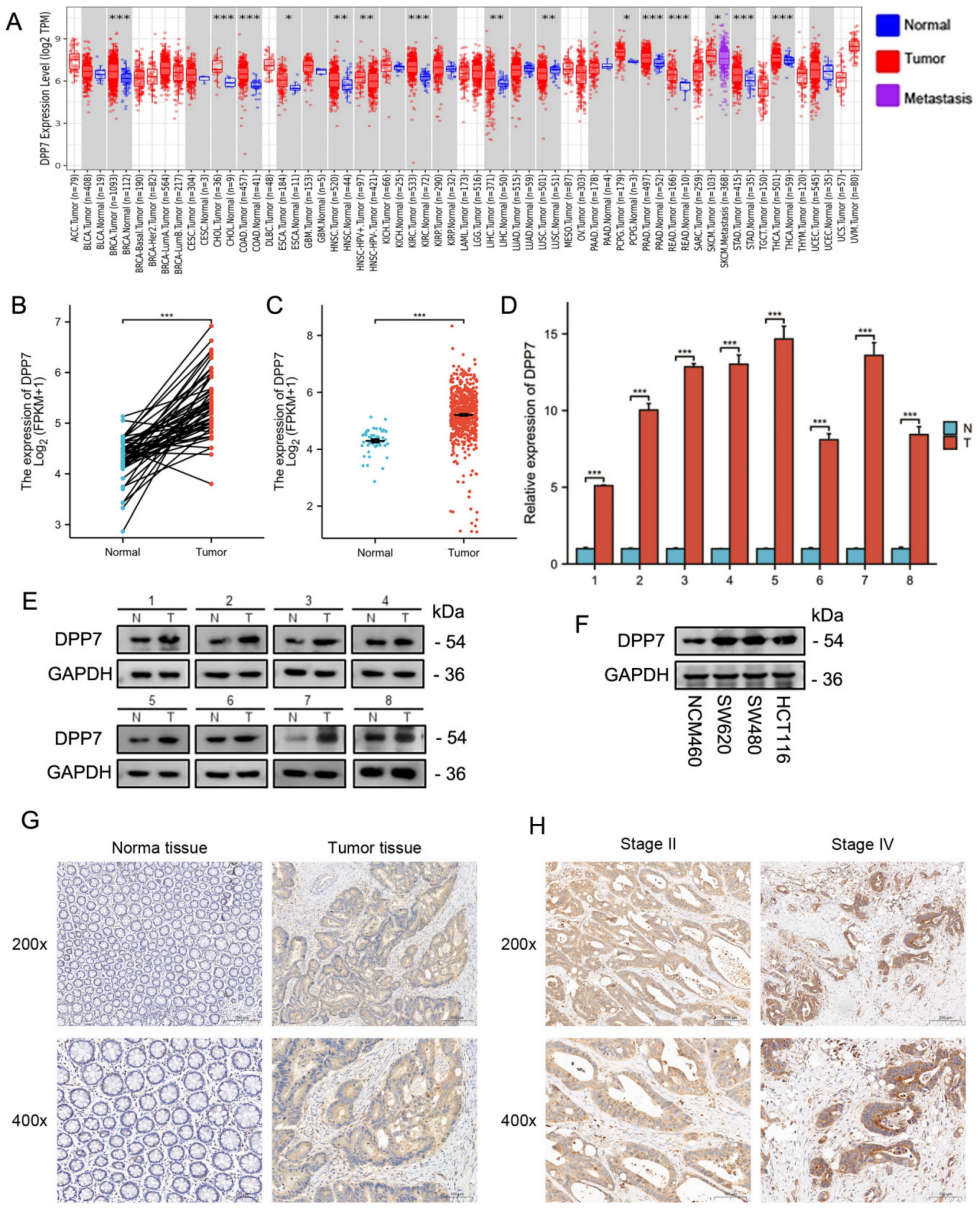
** Expression of DPP7 in CRC. (A)** Comprehensive analysis of DPP7 in tumor. **(B)** Differences in DPP7 expression in colorectal cancer tissues of paired samples in the TCGA database. **(C)** Differences in DPP7 expression in colorectal cancer tissues from unpaired samples in the TCGA database.** (D)** DPP7 expression in CRC tissues examined by qPCR (8 samples). **(E)** DPP7 expression in CRC tissues detected via western blot (8 samples). **(F)** DPP7 expression in CRC cells and normal colorectal epithelial cells examined by western blot. **(G)** IHC staining images of colorectal cancer tissue and adjacent normal tissue.** (H)** IHC staining images of pathological stage II and IV colorectal cancer tissues. *P<0.05, **P<0.01, ***P<0.001.

Through analysis using TIMER2.0, we found that DPP7 is significantly upregulated in various cancer types compared to normal tissues, including but not limited to BRCA, CHOL, COAD, ESCA, HNSC, KIRC, LIHC, LUSC, PCPG, PRAD, READ, SKCM, STAD, and THCA **(Fig. 1A)**. In CRC, we analyzed mRNA expression data for paired samples (from the same patients) **(Fig. 1B)** and unpaired samples **(Fig. 1C)**, revealing a significant upregulation of DPP7 expression in tumor tissues (P < 0.001). The qPCR **(Fig. 1D)** and Western blot **(Fig. 1E)** results from 8 pairs of surgical specimens from the First Affiliated Hospital of Nanchang University also showed increased DPP7 expression in tumor tissues. Furthermore, Western blot analysis indicated a significant upregulation of DPP7 expression in cancer cells such as HCT116, SW480, and SW620 **(Fig. 1F)**. Immunohistochemistry analysis revealed a marked increase in DPP7 expression in colorectal cancer tissues compared to normal colorectal epithelium **(Fig. 1G and Supplementary Fig. 1A)**. These results demonstrate that DPP7 expression is significantly upregulated in CRC at both transcriptional and protein levels.

To determine the correlation between clinical features and DPP7 expression, we grouped patients based on their clinical characteristics and created a table containing clinical baseline data. The clinical baseline data table demonstrates that DPP7 expression is associated with the N stage, lymphatic invasion, and pathological stage** (Supplementary Tables 1 and 2)**. Further analysis of TCGA data and immunohistochemistry results revealed that patients with lymph node metastasis had higher DPP7 expression at transcriptional and protein levels than those without lymph node metastasis** (Supplementary Fig. 1B and 1C)**. Additionally, in CRC specimens with advanced pathological stages, DPP7 expression was higher. Immunohistochemical images of stage II and stage IV specimens are shown here **(Fig. 1H and Supplementary Fig. 1D)**.

### DPP7 is an independent prognostic factor for CRC

**Figure 2:**
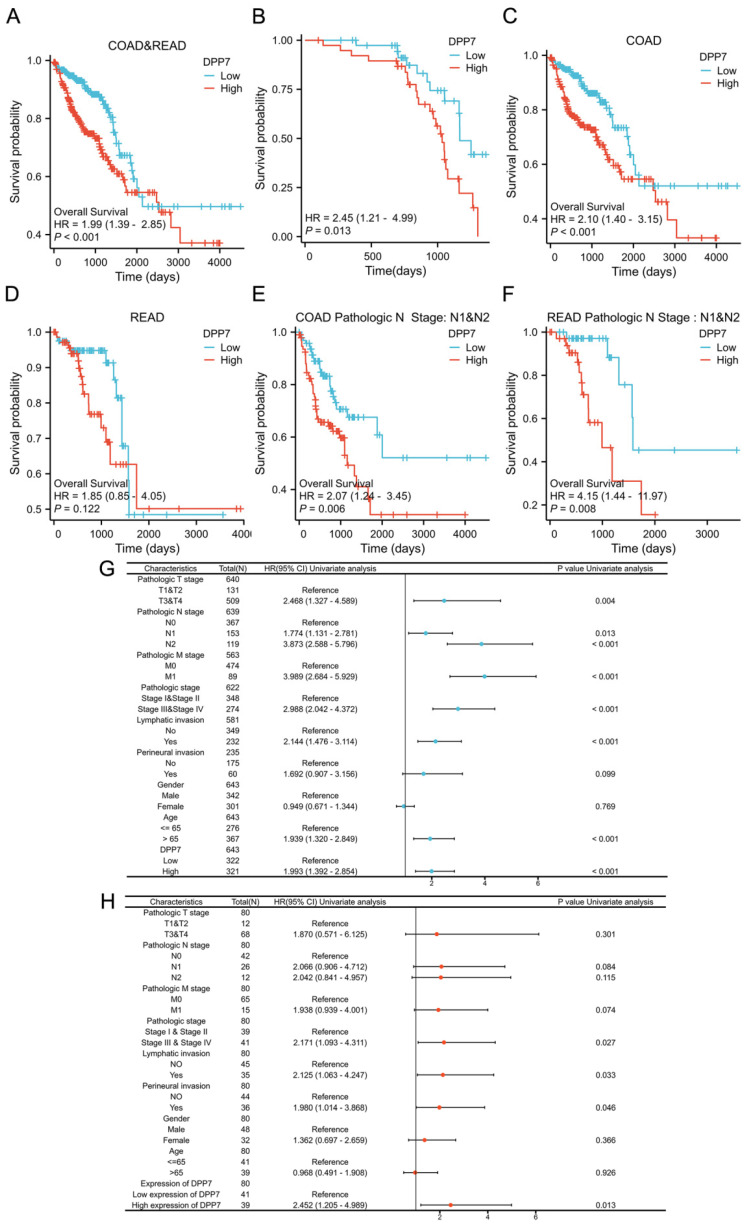
** DPP7 is an independent prognostic factor for CRC. (A)** Prognostic analysis of DPP7 in colorectal cancer in the TCGA database. **(B)** OS analysis of DPP7 in colorectal cancer from IHC results. **(C)** Prognostic analysis of DPP7 in colon cancer in the TCGA database. **(D)** Prognostic analysis of DPP7 in rectal cancer in the TCGA database. **(E)** Prognostic analysis of DPP7 in colon cancer patients with lymph node metastasis in the TCGA database. **(F)** Prognostic analysis of DPP7 in rectal cancer patients with lymph node metastasis in the TCGA database. **(G)** Univariate Cox regression analysis results of DPP7 in colorectal cancer in the TCGA database. **(H)** Univariate Cox regression analysis results of DPP7 in colorectal cancer from IHC results. *P<0.05, **P<0.01, ***P<0.001.

To determine the prognostic value of DPP7 in CRC, we stratified patients into low and high DPP7 expression groups based on the median DPP7 expression from the TCGA dataset for survival analysis. We also validated these findings using immunohistochemistry samples. The results showed that high DPP7 expression was associated with poorer OS in CRC patients** (Fig. 2A and 2B)**. Although survival analysis of patients revealed differences, we observed an overlap in survival outcomes between the high and low DPP7 expression groups in the TCGA dataset. We hypothesized that the location of the tumor in the colon or rectum may influence postoperative survival quality and time. Therefore, we conducted separate analyses for patients with tumors originating from the colon and rectum. We found that patients with high DPP7 expression in colon cancer had worse prognosis, while there was no significant difference in prognosis among rectal cancer patients based on DPP7 expression **(Fig. 2C and 2D)**. However, varying degrees of overlap in survival analysis were still observed in both types of tumors, prompting consideration of potential confounding factors. Previous analyses indicated that patients with lymph node metastasis had higher DPP7 expression than those without metastasis. Therefore, we further stratified colon and rectal cancer patients into subgroups based on lymph node metastasis for analysis. The analysis revealed that patients with high DPP7 expression had worse prognosis in both colon and rectal cancer patients with lymph node metastasis **(Fig. 2E and 2F)**. These findings suggest that DPP7 may be an important prognostic factor for CRC. Additionally, we performed Cox regression analysis on immunohistochemistry and clinical data. Single-factor Cox regression analysis showed that DPP7 was a poor prognostic risk factor, and multivariate regression analysis indicated that DPP7 was an independent poor prognostic risk factor** (Fig. 2G and 2H; Supplementary Table 3 and Supplementary Table 4)**, and these results were validated in the TCGA dataset.

### Identification and enrichment analysis of differentially expressed genes

**Figure 3:**
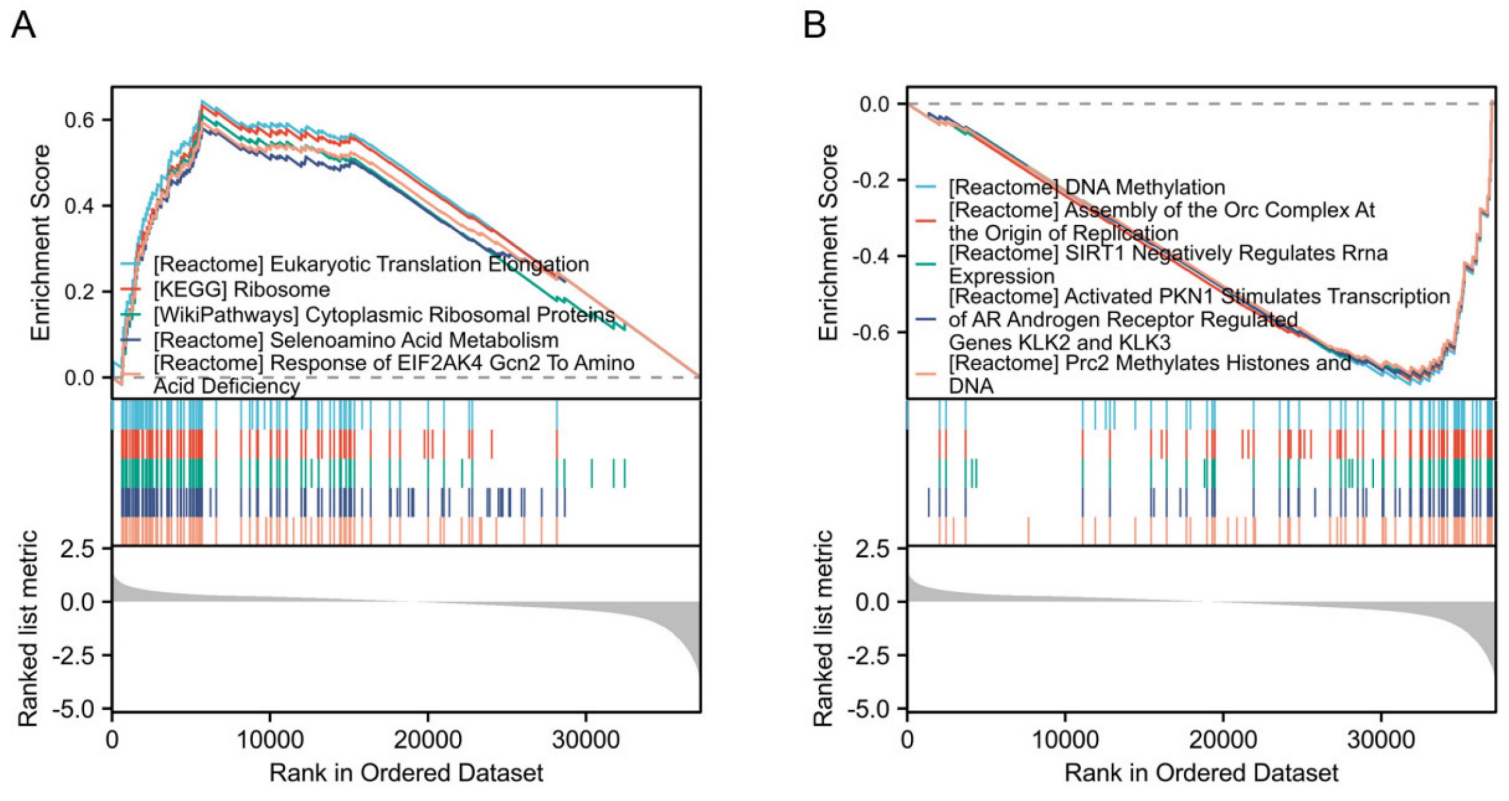
**Enrichment analysis of differentially expressed genes. (A)** Top five GSEA pathways positively correlated with DPP7 expression. **(B)** Top five GSEA pathways negatively correlated with DPP7 expression. *P<0.05, **P<0.01, ***P<0.001.

To identify the signaling pathways regulated by DPP7 abnormal expression, we conducted a comparative analysis between the DPP7 high-expression group and the low-expression group based on the TCGA dataset, using hallmark gene sets. The analysis revealed the top five upregulated pathways, including "Eukaryotic translation elongation," "Ribosome," "Cytoplasmic ribosomal proteins," "Selenoamino acid metabolism," and "Response of EIF2AK4 gcn2 to amino acid deficiency" (**Fig. 3A**). Conversely, the top five significantly downregulated pathways comprised "DNA methylation," "Assembly of the ORC complex at the origin of replication," "Sirt1 negatively regulates rRNA expression," "Activated PKN1 stimulates transcription of AR androgen receptor regulated genes KLK2 and KLK3," and "PRC2 methylates histones and DNA" (**Fig. 3B**).

### Increased expression of DPP7 enhances proliferation, invasion, and migration abilities of colorectal cancer cells

**Figure 4:**
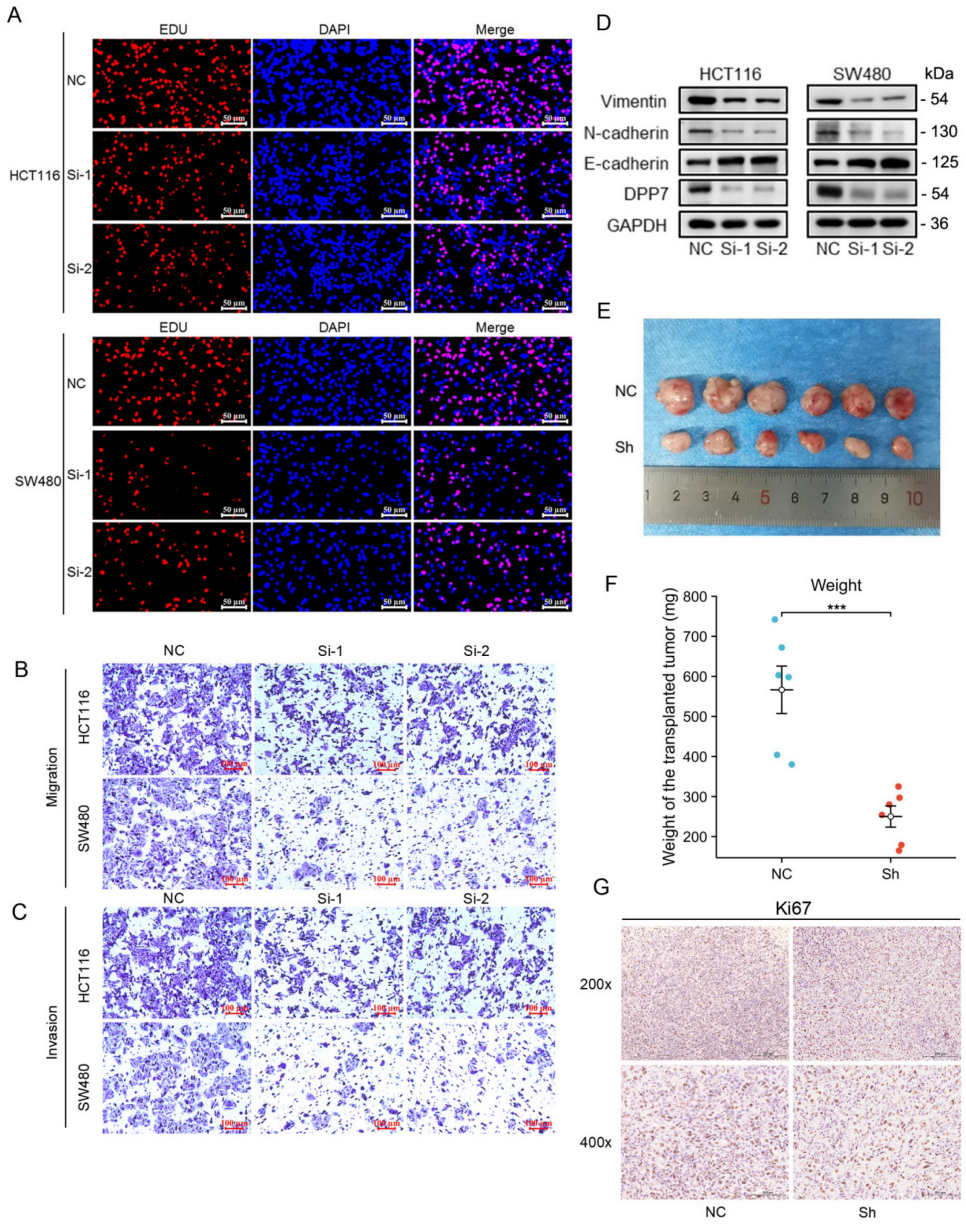
** Increased expression of DPP7 enhances the proliferation, invasion, and migration abilities of colorectal cancer cells. (A)** EdU experiment to detect the effect of knocking down the expression of DPP7 on the proliferation of HCT116 and SW480 cells. **(B)** Transwell chamber migration assay to detect the effects of knocking down the expression of DPP7 on the migration abilities of HCT116 and SW480 cells.** (C)** Transwell chamber invasion assay to detect the effects of knocking down the expression of DPP7 on the invasion abilities of HCT116 and SW480 cells.** (D)** Knockdown of DPP7 expression down-regulates the expression of EMT process-related proteins in colorectal cancer cells. **(E)** Comparison of subcutaneous tumor size between two groups of nude mice after knocking down the expression of DPP7. **(F)** After knocking down the expression of DPP7, the weight of subcutaneous tumors in two groups of nude mice. **(G)** Immunohistochemistry experiment to detect the effect of knocking down DPP7 expression on Ki-67 expression. *P<0.05, **P<0.01, ***P<0.001.

We first used siRNA to interfere with DPP7 expression to observe its effect on the biological behavior of HCT116 and SW480 cells. We found that interference with DPP7 expression could inhibit the proliferation ability of HCT116 and SW480 cells **(Fig. 4 A and Supplementary Fig. 2A-C)**, while overexpression of DPP7 could enhance the proliferation ability of HCT116 and SW480 cells **(Supplementary Fig. 3A and 3B)**. Additionally, in studies of cell invasion and migration, it was found that the knockdown of DPP7 could inhibit the invasion and migration of HCT116 and SW480 cells **(Fig. 4B-C and Supplementary Fig. 2D-E)**. Immunoblotting analysis of epithelial-mesenchymal transition (EMT)-related proteins revealed that downregulation of DPP7 expression led to upregulation of the epithelial marker E-cadherin expression. In contrast, expression of the mesenchymal markers N-cadherin and vimentin were downregulated **(Fig. 4D)**. Overexpression of DPP7, on the other hand, promoted invasion and migration of these two tumor cells **(Supplementary Fig. 3C)**, and the expression of EMT-related proteins was the opposite of that of knockdown of DPP7 **(Supplementary Fig. 4A)**.

Furthermore, we found that the knockdown of DPP7 expression inhibited the growth of SW480 tumors in nude mice subcutaneously **(Fig. 4E and 4F)**. Immunohistochemical staining for Ki67 showed that knockdown of DPP7 resulted in weaker Ki67 staining in the tumors **(Fig. 4G)**.

### DPP7 expression is correlated with immune infiltrating cells and PD-1 expression

Through ssGSEA immune cell infiltration analysis, we observed several correlations between DPP7 expression and specific immune cell types in CRC. DPP7 expression showed a negative correlation with T helper cells, while it exhibited positive correlations with Tregs, cytotoxic cells, NK cells, and DCs **(Supplementary Fig. 5A)**. Furthermore, our analysis of immune checkpoint genes revealed a positive correlation between DPP7 expression and PD-1 and a negative correlation with PD-L1 **(Supplementary Fig. 5B)**. These findings suggest that DPP7 may have associations with the immune response and immune checkpoint regulation in CRC.

### Overexpression of DPP7 suppresses tumor cytotoxicity of Jurkat cells in co-culture

Cytotoxic T cells are the primary immune cells responsible for killing tumors, but their function is often suppressed in the tumor microenvironment^26^, ^27^. However, our analysis of immune cell infiltration revealed a correlation between DPP7 expression in tumor cells and cytotoxic T cells, which seems difficult to comprehend. Therefore, we constructed a co-culture model composed of activated Jurkat cells, HCT116, and SW480 cells to investigate the effect of tumor cells' DPP7 expression on the killing function of activated Jurkat cells.

**Figure 5:**
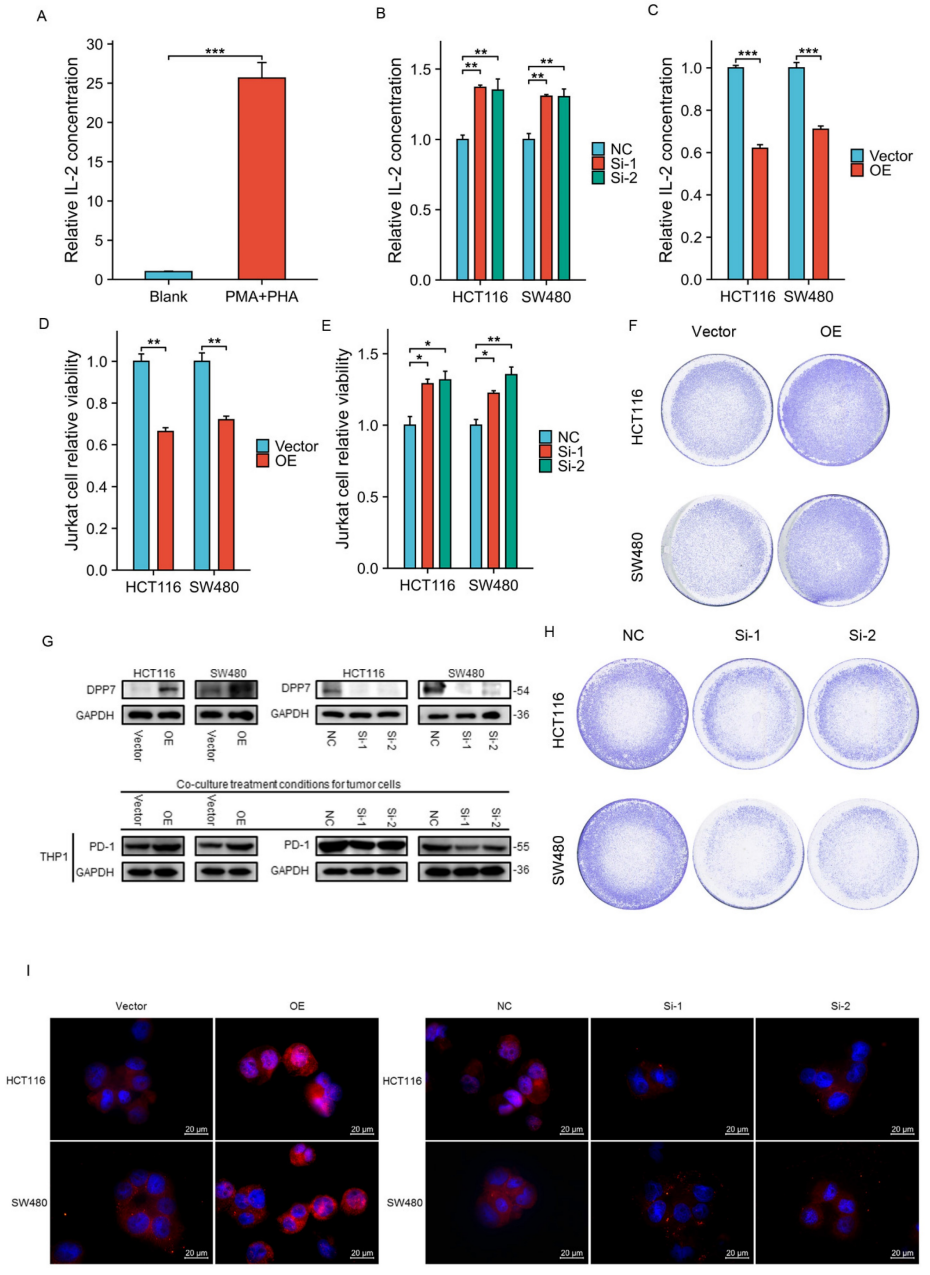
** Co-culture of immune cells and tumor cells. (A)** Relative expression level of IL-2 in Jurkat cells after activation. **(B)** Relative levels of IL-2 in the co-culture system after knocking down DPP7 in tumor cells. **(C)** Relative levels of IL-2 in the co-culture system after overexpressing DPP7 in tumor cells.** (D)** CCK-8 experiment detects the relative viability of Jurkat cells in the co-culture system after overexpressing DPP7 in tumor cells. **(E)** CCK-8 experiment detects the relative viability of Jurkat cells in the co-culture system after knocking down DPP7 in tumor cells. **(F)** Crystal violet staining to observe the surviving tumor cells in the co-culture system after overexpressing DPP7 in tumor cells. **(G)** Western blot experiment to detect the effects of knockdown and overexpression of DPP7 on PD-1 expression in THP-1 cells in the co-culture system. **(H)** Crystal violet staining to observe the surviving tumor cells in the co-culture system after knocking down DPP7 in tumor cells. **(I)** Immunofluorescence experiments were performed to detect the effects of knockdown and overexpression of DPP7 on PD-1 expression in THP-1 cells in the co-culture system. *P<0.05, **P<0.01, ***P<0.001.

Firstly, Jurkat cells were activated using PHA and PMA, and the level of IL-2 in the culture supernatant was measured by ELISA to assess the activation efficiency. The increased secretion of IL-2 by Jurkat cells indicates successful activation** (Fig. 5A)**. IL-2 is a crucial indicator of activated Jurkat cell immune killing, and we will later use it as an indicator of Jurkat cell killing function. We found that the knockdown of DPP7 in tumor cells increased the level of IL-2 in the co-culture system** (Fig. 5B)**, while overexpression of DPP7 decreased the level of IL-2 in the medium** (Fig. 5C)**. Additionally, using CCK-8 assay, we detected the viability of residual tumor cells in the co-culture system and found that knockdown of DPP7 decreased the viability of residual tumor cells compared to the control group, while overexpression of DPP7 improved cell viability compared to the control group** (Supplementary Fig. 4B-C)**. The results of crystal violet staining of residual tumor cells were consistent with the CCK-8 viability results **(Fig. 5F and H)**.

Studies have shown that CD8+ T cells are one of the core forces controlling tumors. However, sustained antigen stimulation can lead to T cell exhaustion, characterized by inefficient immune function and low proliferation capacity^26^, ^28^. We suspected that overexpression of DPP7 in tumor cells would accelerate T-cell exhaustion in the co-culture microenvironment. Therefore, using CCK-8 assay, we detected the viability of Jurkat cells in the co-culture system. The results showed that overexpression of DPP7 led to a more significant decrease in the viability of Jurkat cells in the co-culture system compared to the control group** (Fig.5D)**. In contrast, knockdown of DPP7 resulted in increased viability of Jurkat cells compared to the control group **(Fig.5E)**. In summary, high expression of DPP7 may mediate immune escape by inhibiting the anti-tumor ability of Jurkat cells in the co-culture microenvironment.

### Upregulation of DPP7 in colorectal cancer cells induces increased expression of PD-1 in THP-1 cells

Our analysis of immune checkpoint gene correlation showed that the expression level of DPP7 was positively correlated with the expression level of PD-1. However, in the tumor microenvironment, tumor-associated macrophages (TAMs) are the most abundant immune cells^29^-^31^, and the expression of PD-1 in TAMs gradually increases with tumor progression^32^. Moreover, overexpression of PD-1 in TAMs can inhibit the polarization of macrophages toward the M1 phenotype and support their polarization toward the M2 phenotype, thereby promoting immune escape in tumors. Therefore, we used activated THP-1 cells to investigate the relationship between tumor DPP7 expression and PD-1.

First, we differentiated THP-1 cells into M0 macrophages by activating them with PMA. Then, we extracted samples of activated THP-1 cells from the co-culture system to detect changes in PD-1 expression by immunoblotting and immunofluorescence experiments. The results showed that overexpression of DPP7 in HCT116 and SW480 cells led to increased PD-1 expression in THP-1 cells in the co-culture system at the protein level, while knockdown of DPP7 had the opposite effect** (Fig. 5G and 5I)**.

In summary, high expression of DPP7 in tumor cells increases the expression of PD-1 in activated THP-1 cells in the co-culture system.

## Discussion

Colorectal cancer ranks second in terms of mortality globally, posing significant health risks and economic burdens to humans and society. Some common risk factors for colorectal cancer include familial inheritance, age, nutrition, lifestyle, and environment^33^. Patients often have difficulty detecting the disease early, missing the opportunity for early surgical treatment, thereby affecting prognosis. Research has shown that survival rates for colorectal cancer can be significantly improved through early diagnosis and treatment^34^. Currently, treatment methods for colorectal cancer include surgery, chemotherapy, immunotherapy, gene therapy, and combination therapy, with targeted therapy emerging as a relatively new and promising approach that has been shown to increase OS rates and inhibit cancer cell development. However, the number of available targeted drugs is limited^35^. Therefore, it is crucial to develop and validate new prognostic markers that can effectively predict patient clinical outcomes.

The results of this study indicate that DPP7 is upregulated in various malignant tumors, including colorectal cancer. Transcriptomic analysis and immunohistochemical analysis of clinical samples confirmed elevated DPP7 expression in colorectal cancer, consistent with recent research findings^36^, ^37^. Analysis of clinical characteristics and DPP7 expression collected from the First Affiliated Hospital of Nanchang University revealed that patients with high DPP7 expression had poorer prognosis, consistent with results obtained from the TCGA database. Further analysis of baseline data from TCGA and clinical samples showed that high expression of DPP7 was significantly associated with advanced N stage, advanced pathological stage, and lymphatic invasion. Prognostic data analysis indicated poorer prognosis in patients with lymph node metastasis and high DPP7 expression. Additionally, univariate and multivariate Cox regression analysis revealed that N stage, M stage, pathological stage, age, and DPP7 expression were independent prognostic factors in the TCGA database, while DPP7 expression in clinical samples was an independent prognostic factor. It is speculated that this may be due to differences in ethnicity and sample size between the TCGA database and clinical samples.

*In vitro* cell experiments demonstrated that overexpression of DPP7 enhances the proliferation, invasion, and migration of colorectal cancer cells, while knockdown of DPP7 expression can inhibit tumor cell proliferation, invasion, and migration. Furthermore, immunoblotting experiments revealed that DPP7 affects the expression of EMT-related proteins in colorectal cancer cells, potentially playing an important role in promoting the EMT process of colorectal cancer cells. EMT is an essential process in the development of tumors^38^. It is a triggering factor for tumor cell migration, invasion, and metastasis, as it is associated with reduced cell adhesion and increased motility^39^, ^40^. Several studies have shown a close relationship between the occurrence and development of colorectal cancer and the EMT process^41^, ^42^. Moreover, activation of EMT leads to increased dissemination of colorectal cancer cells and contributes to the development of resistance to chemotherapy and radiotherapy. Targeting EMT or related mechanisms may be a promising approach for the clinical treatment of colorectal cancer patients^43^. Furthermore, the downregulation of DPP7 expression inhibits tumor formation in colorectal cancer. Based on these research findings, this study hypothesizes that DPP7 plays a crucial role in the malignant progression of colorectal cancer.

Furthermore, immune infiltration analysis revealed that DPP7 was positively correlated with various immune infiltrating cells, including Tregs, cytotoxic cells, NK cells, and DCs, while negatively correlated with T helper cells.

Tregs are crucial for maintaining immune homeostasis and preventing autoimmune diseases. On the one hand, Tregs help maintain self-immune tolerance and minimize damage associated with excessive immune responses. On the other hand, they help cancer cells evade immune surveillance, thereby promoting tumor progression and metastasis^44^. Tregs are immunosuppressive cells^44^, and in the presence of r Tregs, tumor progression is faster than in the absence of Tregs^45^. Immunoinhibitory cells promote immune escape in tumors by inducing immune suppression and accumulating in tumors, exerting immunosuppressive effects^46^, ^47^. However, our immune infiltration analysis found a positive correlation between Tregs and DPP7 expression, suggesting that Tregs in the tumor immune microenvironment of colorectal cancer patients may promote tumor progression by inhibiting immune cell function.

Classical dendritic cells (cDCs) are crucial in bridging innate and adaptive immunity. As sentinel cells of the myeloid immune system, cDCs are specialized in detecting invading pathogens and cancer cells. They convert the latter into antigenic peptides recognized by T cells to induce and maintain tumor-specific T cell responses^48^. Cellular factors derived from tumors and the stroma recruit DCs to tumor sites, which may also affect the maturation, differentiation, and function of DCs, leading to insufficient formation of anti-tumor immune responses or the development of DC-mediated tolerance and immune suppression^49^. Although there is abundant infiltration of DCs in tumors, the reality is that the proliferation capacity of T cells induced by DCs in the tumor microenvironment is low. Therefore, DCs in the tumor microenvironment are generally considered immunosuppressive or immunotolerant^50^, ^51^. Thus, DCs associated with high DPP7 expression in colorectal cancer may not promote immunity as expected; instead, they may promote tumor progression similar to the action of Treg cells.

NK cells possess various anti-tumor functions and can limit their activation against non-malignant cells. However, due to NK cells' high energy metabolism requirements, their metabolic status is the primary determinant of their effector function. Tumor cells undergo metabolic reprogramming to adapt to various adverse environments and consume nutrients in the tumor microenvironment. Additionally, tumor cells can secrete metabolites that hinder NK cell anti-tumor function^52^, ^53^. Several studies have reported that NK cells in tumors undergo functional exhaustion and loss, which has been demonstrated in melanoma^54^. The activation of NK cells partly requires the involvement of DCs, which, as discussed earlier, play a role in suppressing immune responses in the tumor microenvironment. Although the results of this study indicate a positive correlation between DPP7 expression and NK cells in the tumor microenvironment, NK cells will face considerable challenges in exerting anti-tumor effects in the tumor microenvironment due to the factors above. Immune infiltration analysis revealed a positive correlation between NK cells and DPP7 expression. Natural killer T (NKT) cells are a type of T cell that can be activated in an HLA-unrestricted manner and exert cytotoxic effects on tumor cell immunity^55^. Therefore, we co-cultured Jurkat cells with tumor cells. Still, the results showed that when DPP7 expression in tumor cells was elevated, the cytotoxic function of activated Jurkat cells was inhibited, leading to decreased secretion of the immune cytotoxicity marker IL-2 on the one hand, and decreased viability of Jurkat cells on the other hand. Consequently, more surviving tumor cells were observed.

T helper cells are core members of adaptive immunity and constitute the last line of defense against pathogen infection and malignant cell invasion by secreting specific cytokines. These cytokines attract or induce the activation and differentiation of other immune cells, including antibody-producing B cells and cytotoxic CD8 T cells. In the tertiary lymphoid structures of tumors, T helper cells provide effective assistance to B cells. In solid organ tumors of non-hematopoietic origin, an increased frequency of follicular T helper cells is typically associated with a better prognosis. The beneficial functions of follicular T helper cells in solid tumors may extend beyond their role in assisting B cells, such as promoting cytotoxic CD8 T cell responses^56^. However, this study's analysis of T helper cells and DPP7 expression revealed a negative correlation between the number of T helper cells and DPP7 expression. T helper cells are immune-active cells, and this result indicates that high expression of DPP7 may inhibit the infiltration of T helper cells, inducing an immune suppressive state.

Furthermore, immunological checkpoint-related gene analysis revealed a positive correlation between PD-1 and DPP7 expression. PD-1 was initially discovered as an immune checkpoint receptor on activated T cells, and its primary function has been widely confirmed to be inhibiting T cell activation after binding to PD-L1^57^. However, it is not only expressed in T cells but also in NK cells, B cells, and macrophages^32^. TAMs are the most abundant immune cells in the tumor microenvironment, and the expression of PD-1 gradually increases with tumor progression. Immunoblotting results showed that upregulation of DPP7 expression could induce increased expression of PD-1 in macrophages in the co-culture system. Studies have reported that overexpression of PD-1 inhibits the phagocytic ability of TAMs towards tumor cells, resulting in severe impairment of macrophage function^58^, ^59^. Additionally, overexpression of PD-1 inhibits the polarization of TAMs towards the M1 phenotype and supports their polarization towards the M2 phenotype, promoting immune escape in tumors^32^, ^60^, while anti-PD-1 can alleviate the immune escape caused by the upregulation of PD-1 expression on TAMs^61^.

Moreover, lymph nodes are crucial sites for the convergence of immune cells for immune signal exchange^44^, ^48^, ^55^. Our analysis of patient prognosis revealed that patients with high DPP7 expression had poorer prognosis in the presence of lymph node metastasis. This may be because high expression of DPP7 by colorectal cancer cells invading lymph nodes severely disrupts the transmission of antigen recognition by immune cells in lymph nodes, leading to immune deficiency in tumors and poorer prognosis for patients.

In summary, elevated expression of DPP7 in colorectal cancer promotes tumor cell proliferation, migration, and invasion on the one hand and mediates immune escape of colorectal cancer by interfering with the immune function of macrophages and Jurkat cells on the other hand. However, this study has limitations in that it did not investigate in depth the mechanisms by which DPP7 regulates the proliferation, migration, and invasion of colorectal cancer cells and the specific mechanisms by which colorectal cancer cells impair the immune function of macrophages and Jurkat cells remain unclear. Nevertheless, this study has revealed the inhibitory effect of overexpressed DPP7 on immune cell function in the colorectal cancer immune microenvironment for the first time, providing insights for future development of anti-colorectal cancer drugs.

## Conclusions

DPP7 is a potential prognostic biomarker for colorectal cancer, and overexpression of DPP7 in colorectal tumors can increase the malignant potential of the tumor and promote immune evasion.

## Supplementary Material

Supplementary figures and tables.
