# Supplementary material for: DPP7 as a Potential Therapeutic Marker for Colorectal Cancer
Source: J Cancer. 2024 Aug 19;15(16):5425–39. doi: 10.7150/jca.93112 (PMC11375546; doi:10.7150/jca.93112)
Supplement: Supplementary file 1 — Supplementary figures and tables. [file jcav15p5425s1.pdf]

Supplementary Fig .1.

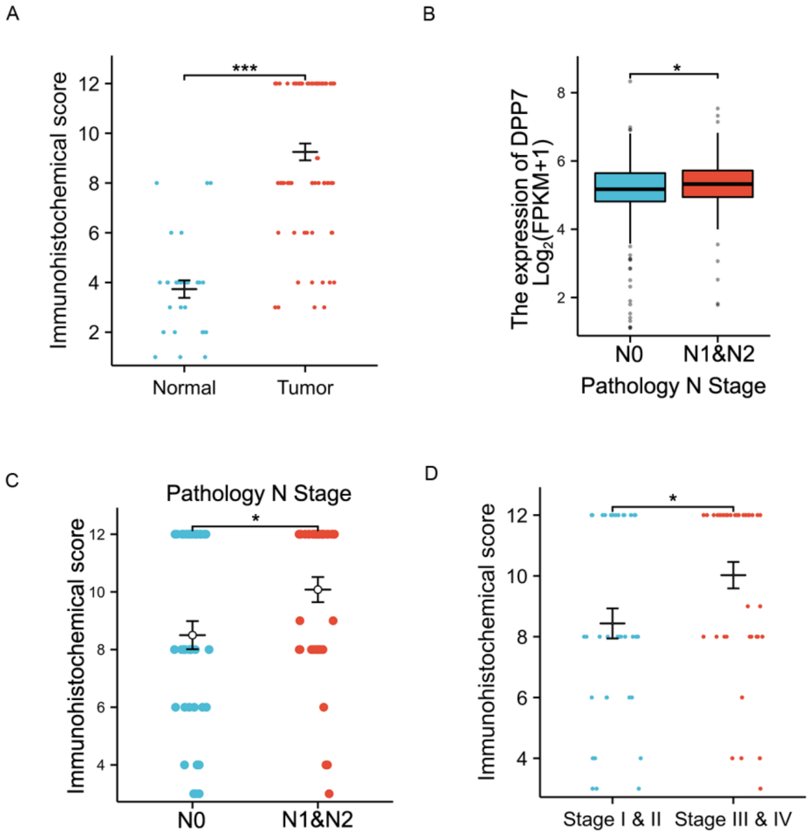

Supplementary Fig .2.

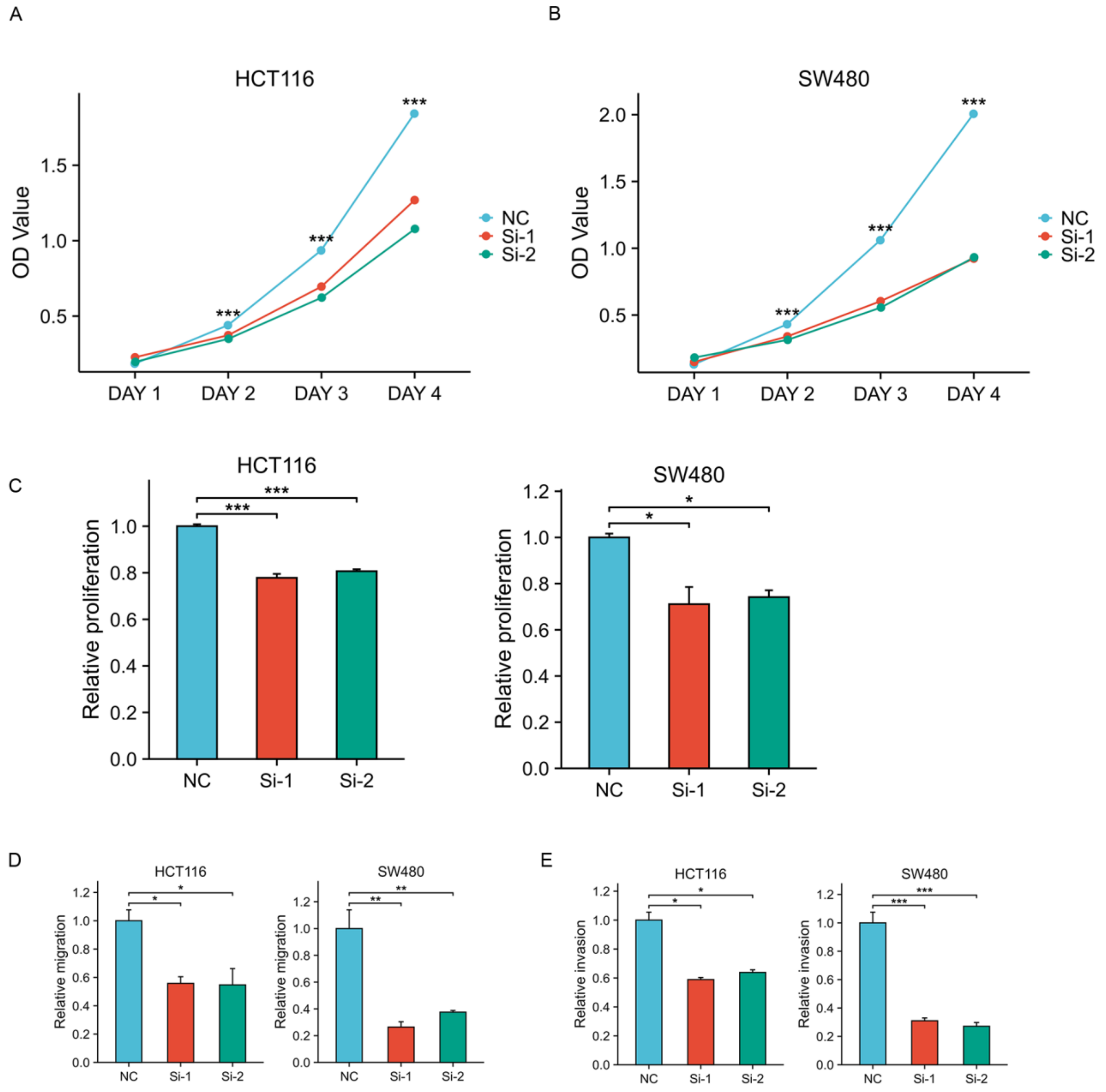

Supplementary Fig .3.

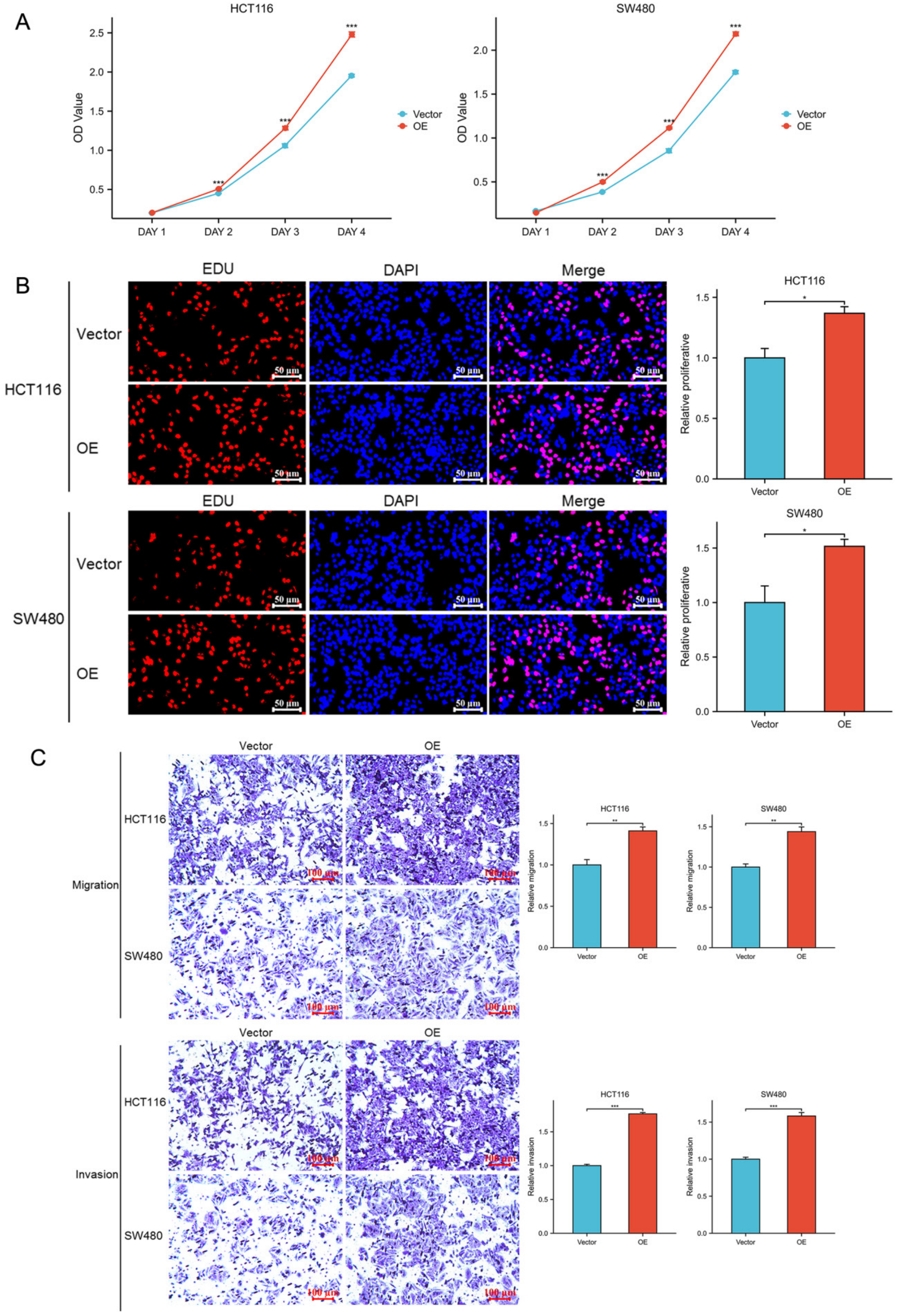

Supplementary Fig .4.

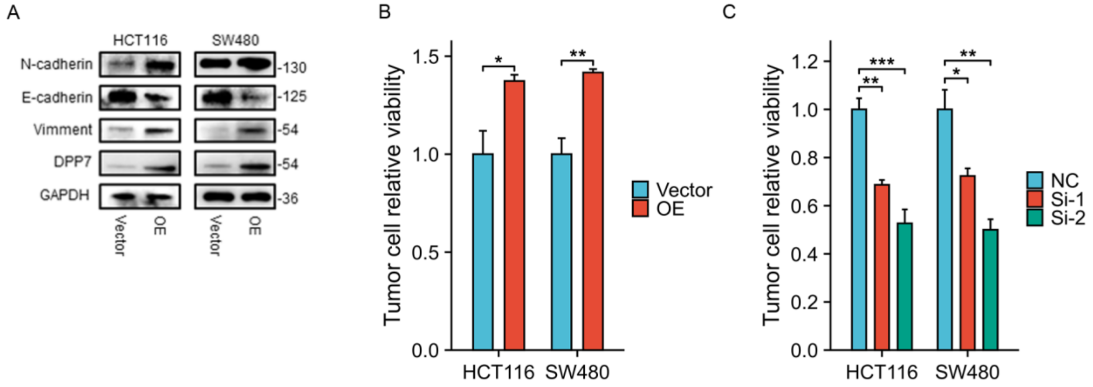

Supplementary Fig .5.

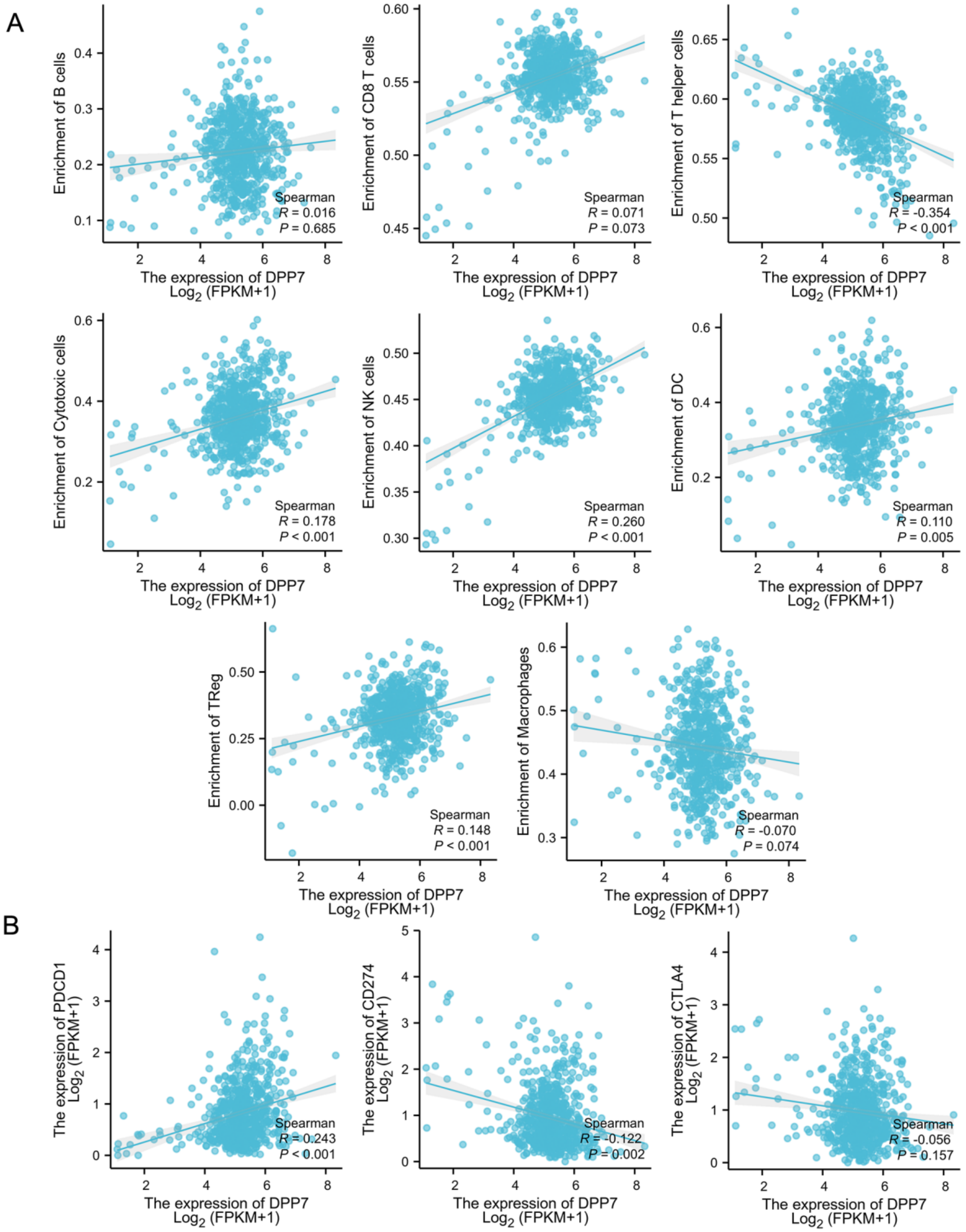

Supplementary Fig.1.

(A) IHC detection of DPP7 in 30 paired CRC tissues. **(B)** Differences in DPP7 expression in tissues of N0 and N1&N2 patients in the TCGA database. **(C)** Differences in DPP7 expression in tissue from patients with stage N0 and stage N1&N2 in IHC results. **(D)** Immunohistochemical detection of DPP7 expression differences in colorectal cancer tissues of pathological stages I&II and III&IV.

Supplementary Fig.2.

(A) CCK-8 experiment to detect the effect of knocking down the expression of DPP7 on the proliferation of HCT116 cells. **(B)** CCK-8 experiment to detect the effect of knocking down the expression of DPP7 on the proliferation of SW480 cells. **(C)** Statistical graph of the effect of knocking down DPP7 expression on the proliferation of HCT116 and SW480 cells was detected by the EdU experiment. **(D)** Migration assay was used to detect the effect of knocking down DPP7 expression on the migration of HCT116 and SW480 cells. **(E)** Invasion assay was used to detect the effect of knocking down DPP7 expression on the invasion of HCT116 and SW480 cells.

Supplementary Fig.3.

(A)CCK-8 detects the effect of overexpression of DPP7 on the proliferation of HCT116 and SW480 cells. **(B)** EdU experiment to detect the effect of overexpression of DPP7 on the proliferation of HCT116 and SW480 cells. **(C)** Transwell chamber migration and invasion experiments were performed to detect the effects of overexpression of DPP7 on the migration and invasion abilities of HCT116 and SW480 cells. *P<0.05, **P<0.01, ***P<0.001.

Supplementary Fig.4.

(A) Overexpression of DPP7 upregulates the expression of EMT process-related proteins in colorectal cancer cells. **(B)** CCK-8 experiment detects the relative viability of tumor cells in the co-culture system after overexpressing DPP7 in tumor cells. **(C)** CCK-8 experiment detects the relative viability of tumor cells in the co-culture system after knocking down DPP7 in tumor cells. *P<0.05, **P<0.01, ***P<0.001.

Supplementary Fig.5.

(A) Relationship between DPP7 expression and eight immune cell types. **(B)** Association of DPP7 expression with PDCD1 (PD-1), CD274 (PD-L1), and CTLA4. *P<0.05, **P<0.01, ***P<0.001.

Supplementary Tables 1

Relationship between DPP7 expression and clinicopathological characteristics in colorectal cancer patients in the TCGA database.

Characteristics	Low expression of DPP7	High expression of DPP7	P value
n	322	322	
Pathologic T stage, n (%)			0.754
T1&T2	67 (10.5%)	64 (10%)	
T3&T4	253 (39.5%)	257 (40.1%)	
Pathologic N stage, n (%)			0.038
N0	195 (30.5%)	173 (27%)	
N1	76 (11.9%)	77 (12%)	
N2	47 (7.3%)	72 (11.2%)	
Pathologic M stage, n (%)			0.110
M0	236 (41.8%)	239 (42.4%)	
M1	36 (6.4%)	53 (9.4%)	
Pathologic stage, n (%)			0.025
Stage I&Stage II	187 (30%)	162 (26%)	
Stage III&Stage IV	122 (19.6%)	152 (24.4%)	
Lymphatic invasion, n (%)			0.002
No	195 (33.5%)	155 (26.6%)	
Yes	99 (17%)	133 (22.9%)	
Perineural invasion, n (%)			0.488
No	96 (40.9%)	79 (33.6%)	
Yes	36 (15.3%)	24 (10.2%)	
Gender, n (%)			0.813
Female	152 (23.6%)	149 (23.1%)	
Male	170 (26.4%)	173 (26.9%)	
Age, n (%)			0.152
<= 65	147 (22.8%)	129 (20%)	
> 65	175 (27.2%)	193 (30%)	

Supplementary Tables 2

Relationship between DPP7 expression and clinicopathological characteristics in colorectal cancer patients in the IHC test results.

Characteristics	Low expression of DPP7	High expression of DPP7	P value
n	41	39	
Pathologic T stage, n (%)			0.925
T3&T4	35 (43.8%)	33 (41.2%)	
T1&T2	6 (7.5%)	6 (7.5%)	
Pathologic N stage, n (%)			0.022
N0	26 (32.5%)	16 (20%)	
N1	13 (16.2%)	13 (16.2%)	
N2	2 (2.5%)	10 (12.5%)	
Pathologic M stage, n (%)			0.334
M0	35 (43.8%)	30 (37.5%)	
M1	6 (7.5%)	9 (11.2%)	
Pathologic stage, n (%)			0.025
Stage I & Stage II	25 (31.2%)	14 (17.5%)	
Stage III & Stage IV	16 (20%)	25 (31.2%)	
Lymphatic invasion, n (%)			0.026
NO	28 (35%)	17 (21.2%)	

Supplementary Tables 2

Relationship between DPP7 expression and clinicopathological characteristics in colorectal cancer patients in the IHC test results.

Yes	13 (16.2%)	22 (27.5%)	
Perineural invasion, n (%)			0.840
Yes	18 (22.5%)	18 (22.5%)	
NO	23 (28.7%)	21 (26.2%)	
Gender, n (%)			0.273
Female	14 (17.5%)	18 (22.5%)	
Male	27 (33.8%)	21 (26.2%)	
Age, n (%)			0.368
<=65	19 (23.8%)	22 (27.5%)	
>65	22 (27.5%)	17 (21.2%)	

Supplementary Tables 3

Univariate and multivariate Cox regression results of colorectal cancer patients in the TCGA database.

Characteristics	Total(N)	Univariate analysis		Multivariate analysis	
		Hazard ratio (95% CI)	P value	Hazard ratio (95% CI)	P value
Pathologic T stage	640				
T1&T2	131	Reference		Reference	
T3&T4	509	2.468 (1.327 - 4.589)	0.004	2.231 (0.928 - 5.366)	0.073
Pathologic N stage	639				
N0	367	Reference		Reference	
N1	153	1.774 (1.131 - 2.781)	0.013	0.263 (0.083 - 0.836)	0.024
N2	119	3.873 (2.588 - 5.796)	< 0.001	0.487 (0.158 - 1.504)	0.211
Pathologic M stage	563				
M0	474	Reference		Reference	
M1	89	3.989 (2.684 - 5.929)	< 0.001	2.092 (1.241 - 3.526)	0.006
Pathologic stage	622				
Stage I&Stage II	348	Reference		Reference	
Stage III&Stage IV	274	2.988 (2.042 - 4.372)	< 0.001	5.975 (1.779 - 20.068)	0.004

Supplementary Tables 3

Univariate and multivariate Cox regression results of colorectal cancer patients in the TCGA database.

Lymphatic invasion	581				
No	349	Reference		Reference	
Yes	232	2.144 (1.476 - 3.114)	< 0.001	1.282 (0.800 - 2.053)	0.302
Perineural invasion	235				
No	175	Reference			
Yes	60	1.692 (0.907 - 3.156)	0.099		
Gender	643				
Male	342	Reference			
Female	301	0.949 (0.671 - 1.344)	0.769		
Age	643				
<= 65	276	Reference		Reference	
> 65	367	1.939 (1.320 - 2.849)	< 0.001	2.733 (1.695 - 4.408)	< 0.001
DPP7	643				
Low	322	Reference		Reference	
High	321	1.993 (1.392 - 2.854)	< 0.001	1.699 (1.100 - 2.623)	0.017

Supplementary Tables 4

Univariate and multivariate Cox regression results of colorectal cancer patients in the IHC test results.

Characteristics	Total(N)	Univariate analysis		Multivariate analysis	
		Hazard ratio (95% CI)	P value	Hazard ratio (95% CI)	P value
Pathologic T stage	80				
T1&T2	12	Reference			
T3&T4	68	1.870 (0.571 - 6.125)	0.301		
Pathologic N stage	80				
N0	42	Reference			
N1	26	2.066 (0.906 - 4.712)	0.084		
N2	12	2.042 (0.841 - 4.957)	0.115		
Pathologic M stage	80				
M0	65	Reference		Reference	
M1	15	1.938 (0.939 - 4.001)	0.074	1.025 (0.404 - 2.601)	0.959
Pathologic stage	80				
Stage I & Stage II	39	Reference		Reference	
StageIII & Stage IV	41	2.171 (1.093 - 4.311)	0.027	1.020 (0.277 - 3.751)	0.976
Lymphatic invasion	80				
NO	45	Reference		Reference	

Supplementary Tables 4

Univariate and multivariate Cox regression results of colorectal cancer patients in the IHC test results.

Characteristics	Total(N)	Univariate analysis		Multivariate analysis	
		Hazard ratio (95% CI)	P value	Hazard ratio (95% CI)	P value
Yes	35	2.125 (1.063 - 4.247)	0.033	1.489 (0.477 - 4.654)	0.493
Perineural invasion	80				
NO	44	Reference		Reference	
Yes	36	1.980 (1.014 - 3.868)	0.046	1.708 (0.818 - 3.568)	0.154
Gender	80				
Male	48	Reference			
Female	32	1.362 (0.697 - 2.659)	0.366		
Age	80				
<=65	41	Reference			
>65	39	0.968 (0.491 - 1.908)	0.926		
Expression of DPP7	80				
Low expression of DPP7	41	Reference		Reference	
High expression of DPP7	39	2.452 (1.205 - 4.989)	0.013	2.307 (1.086 - 4.904)	0.030
